# A label-free nanoplasmonic biosensor for intraoperative discrimination of tumor margins in brain metastases surgery

**DOI:** 10.1007/s11060-026-05683-4

**Published:** 2026-06-27

**Authors:** Víctor García-Milán, Laura Bauluz Olmedo, Sara Marcos, Ruth Lau, Rubén Martín-Láez, Javier Chicote, María Luisa Díaz Fernández, Dennis Céspedes, Dolores Ortiz, Fernando Moreno, José L Fernández-Luna, Alfredo Franco, Carlos Velásquez

**Affiliations:** 1 Department of Neurosurgery, Hospital General Universitario de Elche, Elche, Spain; 2 Instituto de Investigación Marqués de Valdecilla (IDIVAL), Santander, Spain; 3 Department of Neurological Surgery and Spine Unit, Hospital Universitario Marqués de Valdecilla, Av. Valdecilla s/n, Santander, 39008 Spain; 4 Servicio de Anatomía Patológica, Hospital Universitario Marqués de Valdecilla, Santander, Spain; 5 Department of Neurosurgery, Hospital Univeristari Joan XXIII, Tarragona, Spain; 6 Department of Pathological Anatomy, Hospital Universitari Joan XXIII, Tarragona, Spain; 7 Department of Applied Physics, Faculty of Sciences, Universidad de Cantabria, Av. Los Castros, 48, Santander, 39005 Spain; 8 Genetics Unit, Hospital Universitario Marqués de Valdecilla, Santander, Spain; 9 Department of Anatomy and Cell Biology, Universidad de Cantabria, Santander, Spain

**Keywords:** Brain metastases, Plasmonics, Image-guided Surgery, Tumor microenvironment, Neurosurgical procedures.

## Abstract

**Background:**

Brain metastases have traditionally been considered well-demarcated lesions; however, increasing evidence demonstrates frequent microscopic infiltration of the surrounding brain parenchyma, with relevant prognostic implications, despite current intraoperative tools. The aim of this study was to evaluate the feasibility and diagnostic performance of a label-free nanoplasmonic biosensor for intraoperative discrimination between tumor tissue and peritumoral brain in brain metastases surgery.

**Methods:**

A prospective multicenter study was conducted in patients undergoing surgical resection of brain metastases. Paired tumor and adjacent peritumoral tissue samples were collected intraoperatively following a standardized protocol across participating centers. Samples were analyzed ex vivo using a plasmonic nanostructured biosensor, which detects tissue-specific refractive index differences. Histopathological examination served as the reference standard. Paired comparisons were performed using the Wilcoxon signed-rank test, and diagnostic performance was assessed using receiver operating characteristic analysis.

**Results:**

Twenty paired tumor and peritumoral samples from a consecutive series of 20 patients were analyzed. Refractive index values were significantly higher in tumor tissue compared with peritumoral brain (*p* = 0.0008). In 85% of cases, tumor samples showed higher refractive index values than their paired peritumoral counterparts. Using the optimal cut-off value, sensitivity was 76% and specificity was 68%.

**Conclusion:**

Tumor and peritumoral brain tissue can be discriminated through the measurement of intrinsic biophysical properties with a label-free nanoplasmonic biosensor, supporting its potential role as an objective intraoperative tool for margin assessment without the need of exogenous agents.

**Supplementary Information:**

The online version contains supplementary material available at 10.1007/s11060-026-05683-4.

## Introduction

Brain metastases (BM) represent the most common intracranial tumors in adult oncology, with a steadily increasing incidence driven by improved systemic cancer control and longer patient survival [1]. In recent years, prognostic assessment for these patients has become more precise through the use of molecular markers, as reflected in updated scores like the Lung and Melanoma molGPA [2, 3]. Despite advances in systemic therapies and local treatments [4, 5], BM remain a major cause of neurological morbidity [6, 7]. Surgical resection continues to play a key role in selected patients, particularly when gross total resection (GTR) can be achieved [8–10].

Traditionally, BM have been considered well-demarcated and encapsulated lesions, allowing for complete resection based on clear macroscopic boundaries [11]. However, accumulating histopathological and molecular evidence has challenged this paradigm, demonstrating that a substantial proportion of BM exhibit microscopic infiltration into the surrounding brain parenchyma [12–14]. This infiltrative growth pattern complicates the achievement of true GTR, as tumor cells may extend beyond the visually and radiologically defined margins of the lesion.

Current intraoperative tools for margin assessment provide only partial solutions to this problem. Imaging-based techniques, such as intraoperative ultrasound (ioUS) and intraoperative magnetic resonance imaging (ioMRI), provide anatomical guidance but lack biological specificity, require a learning curve, and can be very costly in terms of time and money [15, 16]. Fluorescence-guided surgery and confocal laser endomicroscopy enhance visualization at the tumor boundary [17, 18], yet they rely on exogenous contrast agents and real-time interpretation [19]. As a result, there is still no objective, real-time method capable of characterizing tumor margins based on intrinsic tissue properties.

In this context, nanostructured plasmonic biosensors based on extraordinary optical transmission (EOT) offer a promising label-free technology capable of detecting subtle biophysical differences in tissue [20]. EOT relies on the transmission of light through subwavelength nanohole arrays in metallic films, generating plasmonic resonances that are highly sensitive to local refractive index changes induced by the biological sample in contact with the sensor [21]. This approach has already shown high sensitivity in discriminating glioblastoma from peritumoral brain tissue, based on refractive index (RI) differences, without the need for fluorescent agents or complex tissue processing [22]. In the case of BM, where microscopic infiltration is increasingly recognized as clinically relevant, an EOT-based biosensor may provide complementary information regarding the biophysical characteristics of the tumor–brain interface.

The aim of this study is to assess the intraoperative feasibility and diagnostic performance of a nanostructured plasmonic biosensor for margin assessment in brain metastases surgery by distinguishing tumor tissue from peritumoral brain parenchyma.

## Methods

### Design and subjects

A prospective multicenter study was performed including a consecutive series of patients with BM that were candidates for surgical treatment, treated at Hospital Universitario Marqués de Valdecilla (Santander, Spain) and Hospital Universitari Joan XXIII (Tarragona, Spain). Patients met all the following inclusion criteria: (1) adults *≥* 18 years of age (2) newly diagnosed BM, (3) without prior local treatment, and 3) histopathologically confirmed BM.

Patient selection for surgical treatment was based on the standard clinical protocols and decision-making pathways routinely applied at each participating hospital.

Collection of tissue specimens did not modify the standard of care of the patient, patients’ data were confidentially treated, and research was conducted in accordance with the ethical standards of the Helsinki Declaration. Ethical approval was obtained from the Ethics Committee for Research with Medicines of Cantabria (internal code 2024.285) and from the Comitè Ètic d’Investigació amb Medicaments of the Institut d’Investigació Sanitària Pere Virgili (internal code 024/2026).

###  Clinical and radiological variables

Clinical data were prospectively collected and included patient age, sex, primary tumor origin, and number of brain metastases at diagnosis (solitary or multiple).

Radiological tumor characteristics were assessed using a preoperative plan determined by a semi-automatic manual segmentation process with Brainlab Elements software modules, Cranial 3.0 (Brainlab AG, Munich, Germany) on the preoperative MRI. The collected variables included tumor laterality (right, left, or bilateral), affected lobe (frontal, parietal, temporal, occipital, or cerebellum), number of metastases (solitary or multiple).

### Specimens collection and intraoperative processing

**Fig. 1:**
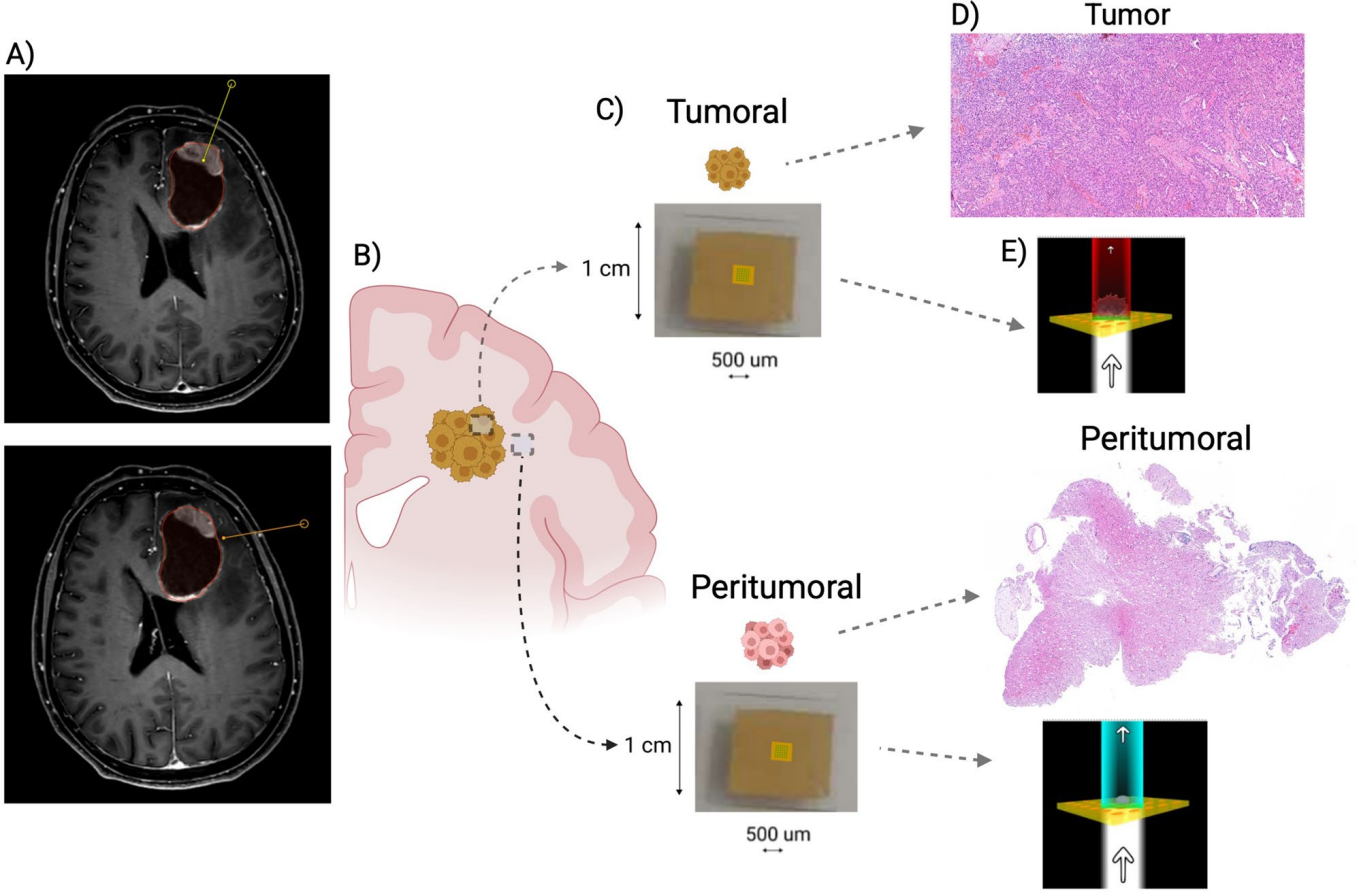
Intraoperative tissue sampling workflow and plasmonic biosensor analysis. (**A**) Representative example of neuronavigation images illustrating the sites of intraoperative tissue sampling. The upper image shows the location where the tumoral sample was obtained, while the lower image indicates the site of peritumoral tissue collection. (**B**) Schematic illustration depicting a brain metastases and the predefined regions selected for tumoral and peritumoral tissue sampling (created with BioRender). (**C**) Macroscopic view of the tumoral and peritumoral tissue specimens deposited onto the surface of the nanoplasmonic biosensor following the standardized intraoperative protocol. (**D**) Representative hematoxylin–eosin staining of the corresponding tumoral and peritumoral samples, confirming tissue classification. (**E**) Schematic plasmonic biosensor readout showing distinct optical responses depending on the tissue imprint. Differences in the transmitted wavelength shift, represented by color variation, reflect differences in the refractive index between tumoral and peritumoral tissue imprints

Tumor and paired adjacent peritumoral tissue specimens were obtained from each case according to a standardized intraoperative tissue-sampling protocol, as detailed in the Supplementary Materials. A schematic overview of the intraoperative workflow, including tissue sampling, processing, and downstream analysis, is shown in Fig. 1.

For the purpose of this study, peritumoral tissue was defined as the brain tissue immediately adjacent to the macroscopic tumor margin that did not fulfill histopathological criteria for overt metastatic tissue. Therefore, this category may encompass a biological spectrum ranging from histologically non-tumoral brain parenchyma to tissue presenting tumor-associated microenvironmental changes or low levels of microscopic infiltration that do not meet the criteria for classification as metastatic tissue.

### Biosensor and optical system

**Fig. 2:**
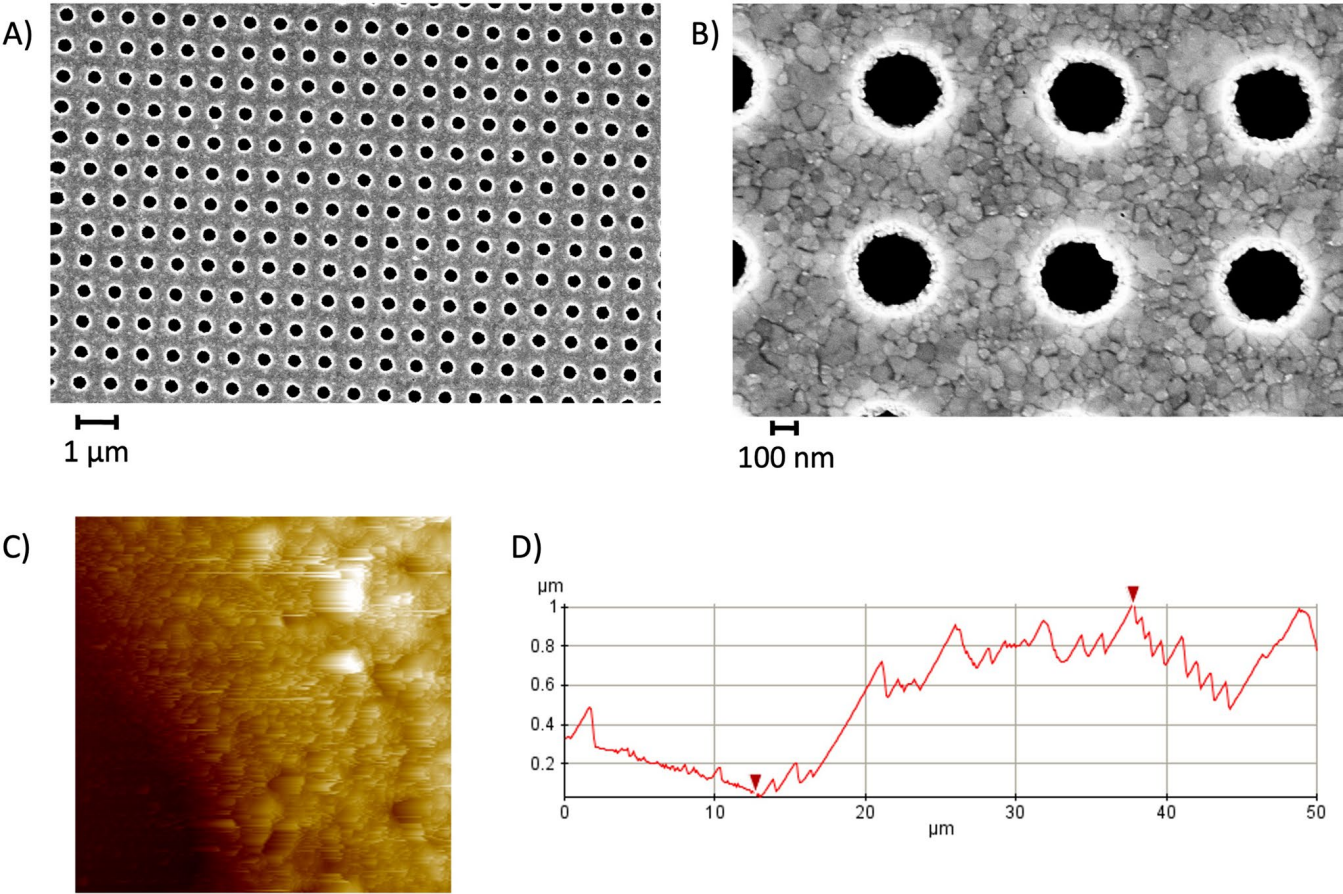
Representative scanning electron microscopy (SEM) and two-dimensional atomic force microscope (AFM) topography images of the nanohole array-based plasmonic biosensor used in this study. (**A**) Picture showing the large-area uniformity of the square array of circular nanoholes patterned on the gold film and the regular periodic distribution of the nanoholes across the sensing surface (magnification: 7190x; voltage: 10.00 kV). (**B**) Picture revealing the circular shape of the nanoholes (magnification: 44810x; voltage: 10.00 kV). **C**) Two-dimensional AFM topography map of a typical print left by a tissue on the biosensor surface. The color contrast represents variations in surface height, where brighter regions correspond to higher elevations and darker regions indicate lower areas or depressions. The print exhibits a non-uniform, textured morphology with visible clusters and ridges distributed across the scanned area. These features suggest variations in the print distribution and its roughness at the microscale. **D**) The graph represents the print thickness variation along a selected line on the sample, revealing a rough and non-uniform distribution of the print on the sensing surface

The main features of the biosensor and its operation have already been described in detail previously [20, 22–24]. The biosensor used for this work consists of a gold film with a square array of circular nanoholes made by electron beam lithography, the nanoholes are distributed periodically along an area of 500 μm x 500 μm (Fig. 2A and B). The nanoholes diameter (340 nm), the array periodicity (780 nm) and the film thickness (80 nm) of the biosensor ensure well-defined spectral peaks and an optimized optical sensitivity in the far red – near infrared region, with typical values within the 450–480 nm/RIU range.

All biosensors were cleaned and characterized before their use. Organic residues were removed from the biosensors surface using plasma and piranha solution as previously reported [20, 22]. The characterization of the biosensors’ optical sensitivity was carried out as described elsewhere [20, 22, 24]. The imprint left by the tissue on the biosensor was analyzed by means of the optical transmission and reflection spectral shifts due to the refractive index (RI) of the imprint. Spectral measurements were performed with the imprint immersed in PBS buffer in regions restricted to a diameter of 400 μm. Both transmission and reflection spectral measurements were performed to ensure the reliability of the results.

The core of the optical system used for the measurements has already been described in detail [20, 24]. Briefly, it is composed of an upright bright field Nikon Eclipse microscope with a 10x objective, an Andor Shamrock 500i spectrograph and a cooled Idus CCD camera coupled to the spectrograph. Both the transmission and reflection spectra were processed and analyzed using in-house software to get the refractive index of each imprint.

### Pathological anatomy

After the imprint process, tissue samples were formalin-fixed and paraffin-embedded for histopathological examination. For each paired tumor and peritumoral sample, two to three histological sections were evaluated using hematoxylin-eosin staining under conventional light microscopy (Fig. 1D). Additional immunohistochemical markers were performed when required to confirm the metastatic origin according to the primary tumor type. Peritumoral samples were classified as non-tumoral when no overt metastatic cells were identified in the examined sections, whereas the presence of metastatic cells within the peritumoral specimen was considered evidence of overt metastatic involvement. In cases with suspected neoplastic infiltration in the peritumoral region, where conventional H&E staining precluded definitive discrimination of isolated tumor cells, an immunohistochemical study for cytokeratins was performed to confirm or rule out their neoplastic nature.

### Statistical analysis

A descriptive statistical analysis was performed to summarize clinical and radiological variables. Continuous variables were summarized as mean ± standard deviation (SD), when normally distributed, or as median and interquartile ranges (IQR) when a non-normal distribution was observed. Categorical variables were reported as absolute counts and percentages. The distribution of continuous variables was assessed using the Shapiro-Wilk test.

Paired comparisons of RI values between tumor and peritumoral tissue samples were performed using the Wilcoxon signed-rank test. Differences were considered statistically significant at a two-sided p-value < 0.05. Considering the paired design of the study, additional paired statistical descriptors were included to further characterize the magnitude and precision of the observed differences between tumor and peritumoral tissue. The effect size (r) associated with the Wilcoxon signed-rank test was calculated to estimate the strength of the paired RI differences. Additionally, the paired refractive index difference (ΔRI, calculated as tumoral RI minus peritumoral RI) was summarized with its corresponding 95% confidence interval obtained using a non-parametric bootstrap approach.

To evaluate the diagnostic performance of the biosensor, a receiver operating characteristic (ROC) curve was constructed. Sensitivity, specificity, and the area under the curve (AUC) with 95% confidence intervals were calculated. The optimal RI cut-off value was determined using the Youden index (J), defined as the point maximizing the difference between true positive and false positive rates (J = sensitivity + specificity – 1) [25].

The statistical analysis was performed using GraphPad Prism version 10.0.0 for Mac OS X (GraphPad Software, San Diego, California USA).

## Results

### Demographics

A total of 20 patients with BM who underwent surgical resection at the participating institutions between July 2024 and February 2026 were initially included in the study. The main clinical, radiological, and demographic features are summarized in Table 1.

**Table 1:** Clinical, sociodemographic and radiological features

CASE	AGE	SEX	SIDE	BM LOCATION	PRIMARY TUMOR ORIGIN	NUMBER OF BM
1	71	Female	Right	Frontal	NSCLC	Solitary
2	79	Male	Right	Parietal	NSCLC	Solitary
3	68	Female	Left	Frontal	Breast carcinoma	Solitary
4	66	Male	Right	Cerebellar	NSCLC	Solitary
5	65	Female	Right	Cerebellar	NSCLC	Solitary
6	62	Male	Right	Cerebellar	NSCLC	Solitary
7	46	Male	Right	Frontal	NSCLC	Solitary
8	69	Male	Right	Frontal	NSCLC	Solitary
9	74	Female	Left	Cerebellar	NSCLC	Multiple
10	57	Male	Right	Cerebellar	NSCLC	Solitary
11	48	Female	Left	Temporal	Breast carcinoma	Multiple
12	64	Female	Right	Temporal	NSCLC	Multiple
13	59	Male	Right	Parietal	Hepatocellular carcinoma	Solitary
14	67	Female	Left	Parietal	NSCLC	Solitary
15	72	Male	Left	Cerebellar	NSCLC	Multiple
16	74	Male	Right	Occipital	Renal cell carcinoma	Solitary
17	74	Male	Right	Cerebellar	NSCLC	Solitary
18	61	Female	Left	Frontal	NSCLC	Solitary
19	70	Male	Left	Cerebellar	NSCLC	Solitary
20	55	Male	Right	Cerebellar	NSCLC	Multiple

Abbreviations: BM = brain metastases; NSCLC = non-small cell lung cancer

The median age at surgery was 66.5 years (range 46–79), and 12 patients were male (60%). Lesions were more frequently located in right hemisphere (13/20, 65%). The most common locations were the cerebellum (9/20, 45%) and the frontal lobe (5/20, 25%), followed by parietal (3/20, 15%) and temporal locations (2/20, 10%). A solitary BM was present in 15 patients (75%), while 5 patients (25%) had multiple intracranial lesions at diagnosis.

Paired tumor/peritumoral samples were analyzed by an expert neuropathologist. All tumor samples were histopathologically confirmed as BM originating from solid tumors. Among them, 16 (80%) corresponded to non-small cell lung cancer (NSCLC), 2 to breast carcinoma (10%), 1 to hepatocellular carcinoma (5%), 1 to renal cell carcinoma (5%). Regarding the paired peritumoral specimens, 19 of 20 samples (95%) were classified as non-tumoral brain tissue, while one case (case 15, 5%) showed tumoral characteristics on histopathological examination.

### Biosensor performance: sensitivity, specificity, and AUC

The RI values obtained by analyzing the imprint on the biosensor are summarized in Table 2. RI values differed significantly between tumor and peritumoral specimens, with higher RI values observed in tumor tissue compared with paired peritumoral brain tissue (Wilcoxon signed-rank test, W = 15, Z = 3.34, *p* = 0.0008). Importantly, the paired comparison showed a large effect size (*r* = 0.75), indicating a strong magnitude of the RI differences between both tissue compartments. The median paired RI difference (ΔRI) was 0.004 RI units (95% CI, 0.003–0.011), further supporting the presence of a consistent optical difference between both tissue types.

**Table 2:** RI values of tumor and peritumoral imprints on the biosensor

CASE	PERITUMORAL RI	TUMORAL RI	ΔRI	PERITUMORAL PATHOLOGICAL ANATOMY*	TUMORAL PATHOLOGICAL ANATOMY*
1	1.360	1.394	+ 0.034	Peritumoral	Metastasis
2	1.345	1.348	+ 0.003	Peritumoral	Metastasis
3	1.345	1.349	+ 0.004	Peritumoral	Metastasis
4	1.349	1.359	+ 0.010	Peritumoral	Metastasis
5	1.345	1.347	+ 0.002	Peritumoral	Metastasis
6	1.352	1.356	+ 0.004	Peritumoral	Metastasis
7	1.346	1.367	+ 0.021	Peritumoral	Metastasis
8	1.340	1.343	+ 0.003	Peritumoral	Metastasis
9	1.347	1.349	+ 0.002	Peritumoral	Metastasis
10	1.342	1.409	+ 0.067	Peritumoral	Metastasis
11	1.355	1.365	+ 0.010	Peritumoral	Metastasis
12	1.340	1.354	+ 0.014	Peritumoral	Metastasis
13	1.336	1.350	+ 0.014	Peritumoral	Metastasis
14	1.338	1.337	-0.001	Peritumoral	Metastasis
15	1.338	1.341	+ 0.003	Metastasis	Metastasis
16	1.361	1.352	-0.009	Peritumoral	Metastasis
17	1.339	1.348	+ 0.009	Peritumoral	Metastasis
18	1.346	1.358	+ 0.012	Peritumoral	Metastasis
19	1.345	1.343	-0.002	Peritumoral	Metastasis
20	1.344	1.347	+ 0.003	Peritumoral	Metastasis

RI, refractive index; ΔRI represents the difference between tumoral and peritumoral RI values calculated as tumor RI minus peritumoral RI. Positive values indicate higher RI in tumor tissue; *Postoperative histopathologic examination

The median RI was 1.350 (IQR 1.347–1.359) in tumor samples and 1.345 (IQR 1.340–1.348) in peritumoral sample. In 17 of 20 cases (85%), RI values were higher in tumor tissue than in the corresponding peritumoral samples. In the remaining 3 cases (15%), the RI difference was negative, with higher RI values observed in peritumoral tissue. Histopathological analysis demonstrated tumoral tissue in one of these peritumoral samples (case 15), whereas the remaining discordant cases showed non-tumoral peritumoral brain tissue.

**Fig. 3:**
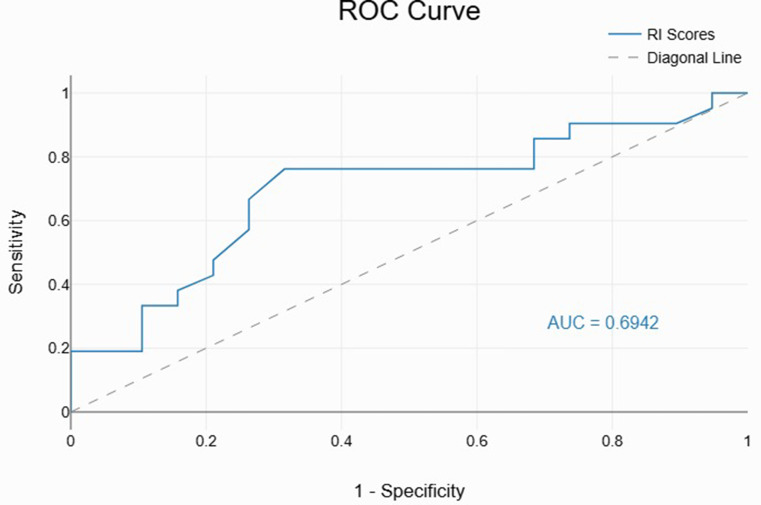
Receiver operating characteristic (ROC) curve for refractive index–based tissue discrimination. The ROC curve illustrates the diagnostic performance of the nanoplasmonic biosensor in distinguishing tumor tissue from paired peritumoral brain tissue based on refractive index (RI) measurements. The solid blue line represents the ROC curve derived from RI values, while the dashed diagonal line indicates the line of no discrimination. The optimal threshold was identified using the Youden index

The diagnostic performance of the biosensor was evaluated using ROC curve analysis. The AUC was 0.6942 (95% confidence interval, 0.5243–0.8641, *p* = 0.02), indicating significant discriminatory ability in distinguishing between the two tissues (Fig. 3). Using the optimal RI cut-off value determined by J (RI = 1.347), the biosensor achieved a sensitivity of 76% (95% CI, 55–89%) and a specificity of 68% (95% CI, 46–85%). At this threshold, the positive predictive value was 72.7%, the negative predictive value was 72.2%, and the overall diagnostic accuracy was 72.5%. These findings demonstrate the ability of the biosensor to detect measurable differences between tumoral and peritumoral tissue; however, the reported diagnostic accuracy should be interpreted as preliminary and requires further validation in larger independent cohorts.

## Discussion

### The tumor–brain interface in brain metastases

BM have traditionally been considered well-demarcated lesions, allowing surgical resection based on visually defined margins. However, accumulating histopathological [26], molecular [14], and imaging evidence [27] has challenged this paradigm, demonstrating that metastatic cells may infiltrate the surrounding brain parenchyma in a substantial proportion of cases [12]. While this infiltrative growth pattern (HGP) has been associated with poorer local control and worse survival [26], highlighting the clinical relevance of accurate margin assessment, recent studies suggest that the HGP serves as a critical surrogate for predicting the specific mechanisms of neurological failure [28].

Recent evidence from the MetInfilt trial and experimental models has shifted our understanding of the tumor–brain interface [29]. As demonstrated by Proescholdt et al., infiltrative HGPs are not only a morphological feature but a critical determinant of survival and a predictor of meningeal metastasis [29]. Furthermore, Komljenovic et al. have elucidated that these infiltrative patterns drive neurological failure through rapid secondary intra-organ dissemination and recolonization by secondary metastasis-initiating cells, whereas non-infiltrative lesions primarily cause death through local mass expansion according to the Monro-Kellie doctrine [28].

In this context, while our study does not aim to quantify the incidence or characteristics of peritumoral infiltration, the nanostructured plasmonic biosensor has proven to be a useful tool in evaluating tumor margins based on intrinsic biophysical properties of the tissue. These findings suggest that RI measurements can capture differences in the biophysical properties of the tumor–brain interface. However, since RI represents a composite physical parameter influenced by multiple biological factors, the present study does not demonstrate the ability to identify microscopic tumor infiltration or detect tumor cells beyond conventional histopathological assessment.

### The discrimination capacity of the biosensor

In this study, we prospectively evaluated the accuracy of a nanoplasmonic tool in providing ex-vivo intraoperative discrimination between BM tissue and the surrounding peritumoral brain. While other technologies have been proposed for near real-time identification of tumor margins in CNS tumors [30, 31], this is the first study to explore the application of an EOT-based system as an intraoperative biosensing tool for real-time delineation of metastatic tissue during surgical resection.

Interestingly, our optical system was able to discriminate between BM and peritumoral tissue without the need for labeling or complex sample preparation, raising the possibility of implementing such technology during surgical resection in the operating room.

Importantly, the main contribution of this proof-of-concept study is not the establishment of a definitive diagnostic classifier, but the demonstration that a label-free nanoplasmonic platform is capable of detecting significant biophysical differences between metastatic tissue and the adjacent brain parenchyma. This observation is reinforced by the paired study design, in which each patient served as their own control, minimizing interindividual variability and demonstrating a large effect size in RI measurements (*r* = 0.75).

The ROC curve analysis (Fig. 3) illustrates the sensitivity and specificity values across different cut-off points of our EOT-based biosensor. In this context, the diagnostic performance observed in our cohort—characterized by a sensitivity of 76% and a specificity of 68%—suggests that although the biosensor can discriminate between tumor and peritumoral tissue above chance level, its current accuracy remains insufficient for independent intraoperative decision-making. Therefore, its potential clinical role should be considered within a multimodal surgical strategy, where plasmonic sensing may provide complementary biophysical information alongside established imaging, fluorescence, or histopathological techniques. Rather than replacing histopathology or existing guidance tools, the biosensor could function as an additional layer of objective information to support maximal safe GTR.

Although the current study relied on an ex vivo imprint-based workflow with laboratory optical analysis, recent developments from our group have demonstrated the feasibility of real-time plasmonic measurements using a reflection-based nanohole sensor configuration [32]. However, further development of a dedicated surgical prototype is required to address challenges related to reproducibility under operative conditions, blood contamination, tissue handling, sterility, and workflow integration.

These findings are consistent with previous results from our group using the same EOT-based nanoplasmonic platform in glioblastoma (GBM), where we reported a sensitivity of 84% and a specificity of 83% in discriminating tumor from peritumoral tissue [22]. Taken together, these data suggest that the biosensor may provide robust real-time feedback on tissue composition across different tumor types and may help guide more accurate resections in BM surgery.

When comparing RI values across tumor types, the median RI values observed in BM and GBM appear broadly similar. In our previous GBM cohort, the median peritumoral RI was 1.341, while the median tumoral RI was 1.350. In the present BM series, median RI values were 1.345 in peritumoral tissue and 1.350 in tumoral samples. Despite these comparable absolute values, important biological differences between both tumor entities may significantly influence RI measurements.

GBM is characterized by a highly infiltrative growth pattern, rapid cellular proliferation, and frequent necrosis, resulting in a complex and heterogeneous microenvironment with pronounced gradients in cellular density, extracellular matrix remodeling, and water content. In contrast, BM typically display a more circumscribed macroscopic architecture, although microscopic infiltration is heterogeneous and often focal. Differences in tumor–host interaction, vascular permeability, inflammatory response, and stromal composition between primary and secondary brain tumors may therefore modulate RI values independently of their absolute magnitude.

Taken together, these observations suggest that similar median RI values across tumor types do not necessarily reflect identical underlying biology. Rather, RI measurements likely integrate multiple microenvironmental and tumor-specific features, which may differ substantially between glioblastoma and BM despite overlapping optical ranges.

However, in three cases, RI measurements showed overlapping values between tumor and paired peritumoral specimens, whereas histopathological analysis confirmed that the paired specimens corresponded to biologically different tissues. These discordant results highlight the limitations of refractive index–based discrimination and underscore the complexity of the tumor–brain interface in BM.

To better interpret these discordant findings, it is important to consider the type of biological information captured by the biosensor. RI is a composite biophysical parameter influenced by multiple tissue features, including cellularity, nuclear-to-cytoplasmic ratio, extracellular matrix composition, lipid and protein content, and water distribution. Consequently, the biosensor does not detect tumor tissue per se, but rather reflects emergent physical properties arising from the underlying tissue microenvironment.

From this perspective, the observed discrepancies may be explained by biological heterogeneity at the tumor–brain interface. In some cases, peritumoral tissue classified as non-tumoral on routine histology may still present microstructural or biochemical alterations—such as reactive changes, edema, gliosis, or focal microscopic infiltration outside the sampled area—that modify its RI. Conversely, tumoral samples with lower cellular density or necrotic components may exhibit RI values closer to those of adjacent brain tissue. Importantly, these findings are more likely to reflect biological variability and sampling-related factors than technical failure of the biosensor.

###  Contextualizing the plasmonic biosensor among current intraoperative tools

Fluorescence-guided surgery with 5-ALA or SF is increasingly used in neuro-oncological procedures [33, 34], as it has been shown to improve the extent of resection and is associated with longer progression-free and overall survival in high grade gliomas [35]. However, these agents require preoperative administration, and in the case of 5-ALA, postoperative precautions are needed due to photosensitivity [36]. Moreover, fluorescence at the tumor margins is often faint and difficult to interpret [37]. In contrast, our nanoplasmonic biosensor is a label-free technology that does not require the administration of any drug to the patient, eliminating the need for preoperative preparation and postoperative care. It operates by detecting subtle biophysical differences between tumor and peritumoral tissue and this could potentially be performed in real time, offering objective, and reproducible data. By removing the subjectivity inherent to fluorescence interpretation, the biosensor provides consistent and biologically relevant information that can support surgical decision-making throughout the entire procedure.

Similarly, CLE is a fluorescence-based technique that allows high-resolution, in vivo visualization of cellular structures during neurosurgery. Its utility is supported by numerous studies [31, 38], and it enables targeted imaging of tumor tissue, the peritumoral region, and their interface. However, CLE requires the systemic administration of SF and relies on the real-time interpretation of the images by an experienced neuropathologist—conditions that may not be feasible in all surgical environments. Moreover, both fluorescence-guided surgery and CLE are fluorescent label-based methods, providing less reliable and reproducible results, and can alter the intrinsic properties of the biological sample studied [39]. In contrast, our plasmonic biosensor is a label-free method that does not require prior drug administration or expert interpretation. It provides immediate, quantitative data based on intrinsic tissue properties, allowing for objective differentiation between tumor and non-tumoral brain tissue.

Fluorescence-guided surgery and CLE currently represent the most advanced intraoperative approaches for assessing the tumor–brain interface in BM. Recent prospective studies, including the MetInfilt trial, have demonstrated the feasibility of combining MRI-guided targeting, fluorescence-assisted surgery, and CONVIVO imaging to identify and histologically characterize infiltrative growth patterns at the metastatic margin [29]. In contrast, the present nanoplasmonic biosensor does not provide direct visualization of tumor cells and represents an earlier-stage technology. Rather than replacing these established approaches, its potential role may lie in providing complementary label-free information based on the intrinsic biophysical properties of the tissue microenvironment.

There are other image-based systems, such as preoperative MRI-based neuronavigation, ioUS, and ioMRI. Neuronavigation provides anatomical orientation during surgery but does not offer biological information about tissue composition and is affected by brain shift as surgery progresses [40, 41]. ioUS provides real-time feedback, but it has a high user dependency and requires a long learning curve and a complex interpretation of results [15, 42]. Lastly, ioMRI can improve the extent of resection by identifying residual tumors during glioma surgery. However, the process of introducing and maintaining iMRI is very costly and time-consuming, without real-time feedback [16, 43, 44] and its use may also increase complications due to prolonged operating time [45, 46].

Overall, the nanoplasmonic biosensor shows a strong capacity to discriminate between tumor and peritumoral tissue. Whether used independently or alongside other intraoperative techniques, it has the potential to be integrated into the surgical workflow for the treatment of BM.

### Limitations

This study has several limitations. First, the small sample size limits the statistical power and precludes extensive subgroup analyses according to primary tumor origin, anatomical location, or molecular profile. Although the paired design strengthens the internal validity of the RI comparisons, larger multicenter cohorts will be required to confirm the robustness and generalizability of these findings across different metastatic entities and surgical scenarios.

Second, the current analysis was performed using an ex vivo imprint-based approach. While this strategy allowed standardized measurements and direct histopathological correlation, it does not yet fully replicate the complexity of the intraoperative environment. Tissue handling, local heterogeneity, and sampling depth may influence the optical signal, and these factors should be further explored in future experimental and clinical studies.

Third, although the biosensor demonstrated statistically significant differences between tumoral and peritumoral tissue, its current diagnostic performance remains moderate. The observed sensitivity and specificity indicate that false-positive and false-negative classifications may occur at a frequency that precludes its use as a standalone intraoperative decision-making tool. This limitation is particularly relevant in the context of BM surgery, where false-positive results could potentially lead to unnecessary resection of functional brain tissue, while false-negative results may contribute to residual microscopic disease. Therefore, the current findings should be interpreted as a proof-of-concept demonstrating the feasibility of detecting tissue-specific biophysical differences rather than evidence supporting immediate clinical implementation. Furthermore, the ultimate clinical utility of plasmonic sensing will likely rely on its integration with other intraoperative modalities, providing complementary information within a multimodal strategy for maximal safe tumor resection.

## Conclusion

The nanoplasmonic biosensor evaluated in this study demonstrated the ability to discriminate between tumor and peritumoral brain tissue ex vivo in patients with brain metastases, based on label-free, real-time refractive index measurements. This system requires no exogenous agents, does not alter tissue integrity, and eliminates subjective interpretation, offering a practical and objective tool for intraoperative use.

## Supplementary Information

Below is the link to the electronic supplementary material.

Supplementary Material 1

## Data Availability

No datasets were generated or analysed during the current study.
