# Supplementary material for: A label-free nanoplasmonic biosensor for intraoperative discrimination of tumor margins in brain metastases surgery
Source: J Neurooncol. 2026 Jun 27;178(3):81. doi: 10.1007/s11060-026-05683-4 (PMC13315513; doi:10.1007/s11060-026-05683-4)
Supplement: Supplementary file 1 — Supplementary Material 1 [file 11060_2026_5683_MOESM1_ESM.docx]

**Supplementary Protocol for Intraoperative Tissue Sampling and Processing for Nanoplasmonic Biosensor Analysis in Brain Surgery**

1. **Preoperative preparation.**

Prior to surgery, all patients were given detailed information about the study's objectives and procedures. Informed consent was obtained for the collection and use of surgical tissue samples for research purposes. Sampling of tissue was performed exclusively for research purposes and did not interfere with standard surgical decision-making or patient care.

Preoperative surgical planning was conducted using MRI-based neuronavigation (Brainlab®, Munich, Germany). The tumor location, planned surgical corridor and potential sampling areas were reviewed prior to surgery. Particular attention was paid to identifying regions suitable for taking paired samples of tumor tissue and adjacent peritumoral brain tissue.

No additional preoperative preparation or medication was required for the use of the nanoplasmonic biosensor. The study protocol did not require administration of exogenous contrast agents or modification of the standard anesthetic or surgical workflow.

1. **Operating room setup and intraoperative workflow.**

On the day of surgery, the operating room workflow followed standard neurosurgical procedures. The study protocol did not require any modification of the surgical setup or interfere with routine patient management.

A dedicated auxiliary table was prepared in a peripheral area of the operating room for tissue handling and sample processing. This table was equipped with sterile forceps, sterile containers, saline solution, and the nanoplasmonic biosensor chips. All materials required for sample manipulation were prepared in advance to minimize handling time and avoid delays during the surgical procedure.

The biosensor analysis was performed ex vivo and did not require placement of any additional equipment within the sterile surgical field. Tissue samples were removed from the sterile field immediately after extraction and transferred to the auxiliary table for processing.

1. **Intraoperative tissue sampling.**

Tissue sampling was performed intraoperatively during craniotomy for tumor resection or biopsy. For each patient, two paired tissue samples were obtained: one tumor sample and one peritumoral brain tissue sample. Sample collection was carried out exclusively when it did not compromise surgical safety or the standard of care. The intraoperative sampling strategy for tumor and peritumoral tissue in cortical and subcortical lesions is schematically illustrated in **Figure S1**.

In the present cohort, 9 lesions were classified as cortical and 11 as subcortical, requiring the corresponding sampling strategy described below.

- 1. **Tumor tissue sampling**

The tumor sample was obtained from the solid portion of the lesion corresponding to the contrast-enhancing area on preoperative MRI. Areas of macroscopic necrosis, extensive hemorrhage, or predominant neovascularization were avoided whenever possible.

Tumor tissue was collected after tumor exposure and initial debulking, using standard microsurgical instruments. The sample was taken from a representative region of viable tumor tissue, ensuring adequate cellularity for subsequent biosensor analysis and histopathological confirmation.


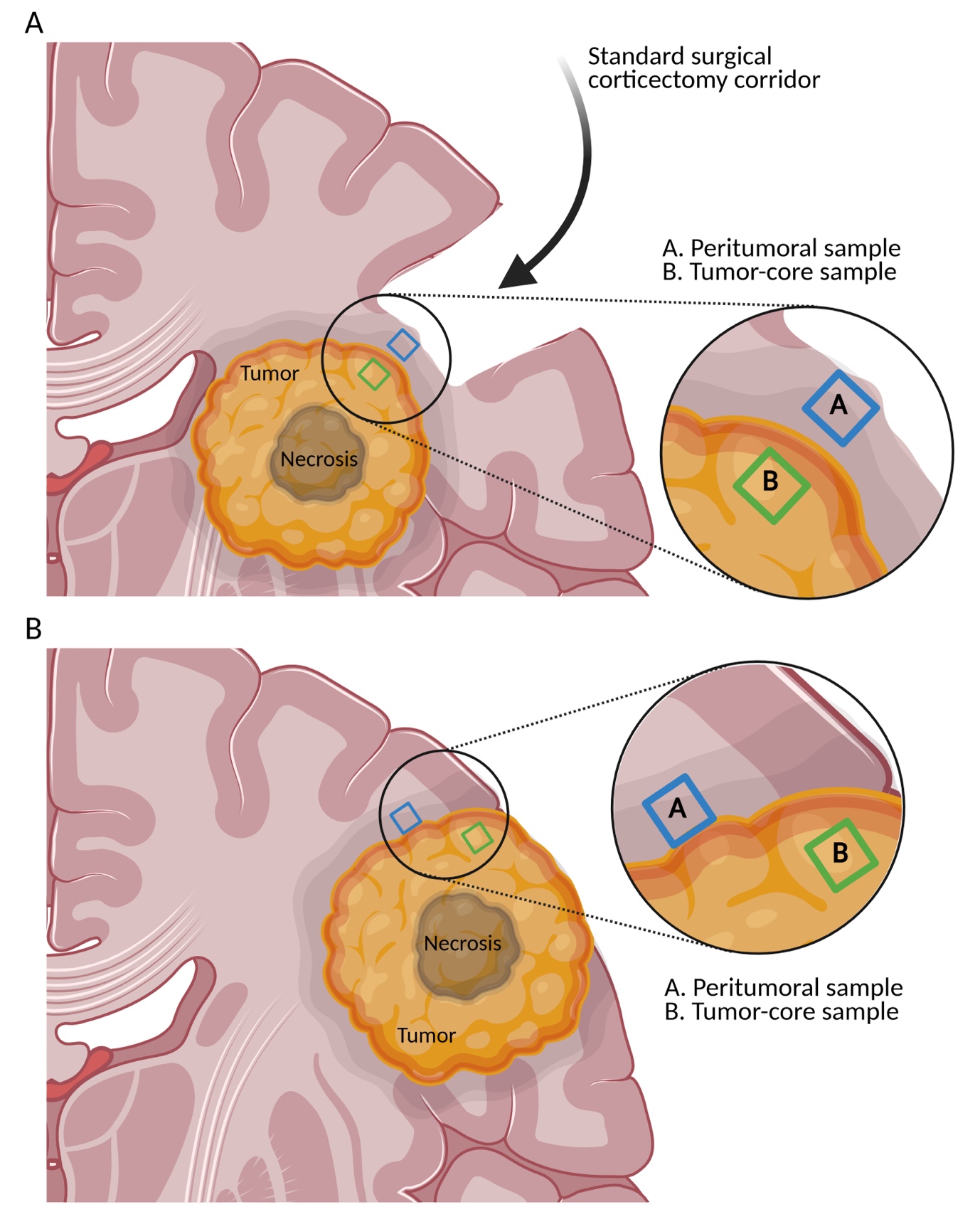


**Figure S1**. Schematic representation of intraoperative tissue sampling strategy in subcortical and cortical brain metastases. (A) In subcortical lesions, peritumoral tissue (A) is sampled from normal-appearing brain parenchyma encountered along the standard corticectomy corridor immediately before tumor exposure, whereas tumor tissue (B) is obtained from the tumor core, avoiding necrotic areas. (B) In cortical lesions (right panel), peritumoral tissue (A) is collected from the resection cavity wall at a non-eloquent site, while tumor tissue (B) is sampled from viable tumor regions. Areas of macroscopic necrosis are systematically avoided in all cases.

- 1. **Peritumoral brain tissue sample**

The peritumoral sample was obtained from brain parenchyma immediately adjacent to the tumor, with a normal macroscopic appearance. The sampling strategy depended on tumor location:

- Subcortical lesions:

Peritumoral tissue was collected from the normal-appearing brain parenchyma encountered immediately before reaching the tumor during the corticectomy along the surgical corridor.

- Cortical lesions:

Peritumoral tissue was obtained from the wall of the resection cavity at a site judged to be non-eloquent and at low risk for functional deficit. Cortical tissue was systematically avoided.

In all cases, sampling was performed under neuronavigation guidance to ensure accurate spatial correlation with preoperative imaging.

- 1. **Sample handling and documentation**

Each tissue sample measured approximately 2–5 mm in maximum diameter. Tumor and peritumoral samples were placed in separate sterile containers containing saline solution and immediately removed from the sterile surgical field for ex vivo processing.

The exact coordinates of each sampling site were recorded using the neuronavigation system. Intraoperative screenshots were captured to document the precise anatomical location of both tumor and peritumoral samples. All images were anonymized and stored for subsequent analysis and correlation.

1. **Ex vivo sample processing and biosensor imprinting.**

All tissue samples were processed ex vivo immediately after intraoperative collection. Once removed from the sterile surgical field, tumor and peritumoral samples were handled separately to avoid cross-contamination.

Each sample was gently rinsed with sterile saline solution to remove excess blood and debris, taking care not to mechanically disrupt the tissue surface. No fixation, staining, or additional preparation was performed prior to biosensor imprint.

For biosensor imprinting, the tissue sample was placed directly onto the surface of the corresponding nanoplasmonic biosensor chip (tumor or peritumoral). The sample was positioned over the central region of the biosensor, ensuring complete coverage of the active sensing area. The tissue surface placed in contact with the biosensor corresponded to the external surface of the sample, without additional compression.

The tissue was left in contact with the biosensor surface for approximately 1 minute. During this time, a member of the research team monitored the contact duration to ensure consistency across samples. This contact allowed the formation of a tissue imprint on the biosensor surface, reflecting the local biophysical properties of the sample. The active sensing area of the nanoplasmonic biosensor consists of a central nanohole array with a surface of 500 × 500 μm, which defines the region used for tissue imprinting and optical measurements (**Figure S2**).


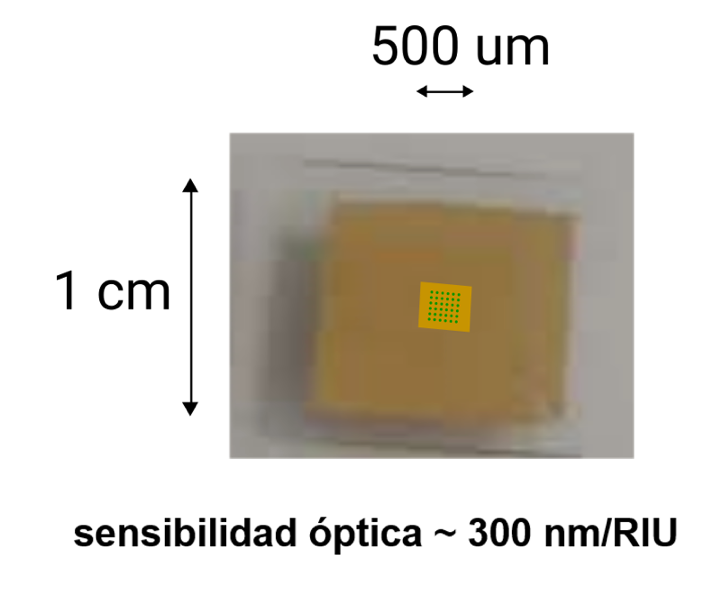


**Figure S2**. Nanoplasmonic biosensor chip and active sensing area. Representative image of the nanoplasmonic biosensor chip (overall size approximately 1 cm), highlighting the central nanohole array that constitutes the active sensing area (500 × 500 μm). The biosensor exhibits an optical sensitivity of approximately 450–480 nm per refractive index unit (nm/RIU), enabling enhanced detection of subtle refractive index variations induced by tissue imprints. This improved sensitivity reflects the optimized nanoplasmonic design used in the current study.

In cases in which the biosensor chip was integrated into a reflection-based measurement device, the tissue sample was placed directly onto the mounted biosensor and analyzed immediately using extraordinary optical transmission–based nanoplasmonic sensing.

After completion of the imprinting process, the tissue sample was carefully removed using sterile forceps. Each sample was then placed in an individual container with formalin and sent for standard histopathological examination to confirm tissue classification (tumor or peritumoral).

Following tissue removal, the biosensor surface was allowed to air-dry for approximately 5–10 minutes to permit evaporation of residual fluid. The biosensors were subsequently stored at 4 °C until optical analysis.

1. **Nanoplasmonic biosensor analysis and optical measurements.**

Optical analysis of the tissue imprints left on the nanoplasmonic biosensor was performed using extraordinary optical transmission (EOT)–based sensing. Measurements were conducted either in transmission or in reflection mode, depending on the experimental configuration, without modifying the tissue imprint or the biosensor surface.

- 1. **Transmission and reflection measurements modes**

In the transmission configuration, broadband light was directed perpendicularly through the biosensor, and the transmitted spectrum was collected below the nanohole array (**Figure S3A**). Changes in the local RI induced by the tissue imprint produced a wavelength shift of the EOT resonance peak, which was quantified for each measurement.

In the reflection configuration, the biosensor was integrated into a reflection-based optical device. In this setup, the incident light interacted with the nanohole array at an oblique angle, and the reflected signal was collected and analyzed (**Figure S3B**). Although the optical geometry differs from the transmission mode, both configurations rely on the same physical principle, namely the sensitivity of the EOT resonance to refractive index variations at the biosensor surface. The choice of configuration did not alter sample preparation or imprinting procedures.

- 1. **Spatial scanning and heat map generation**

For each tissue imprint, optical measurements were performed by scanning the active sensing area of the biosensor. The nanohole array surface (500 × 500 μm) was subdivided into a grid of discrete measurement points, and spectra were acquired sequentially across the imprint.

At each position, the wavelength shift of the EOT resonance was calculated and converted into a local RI value. These spatially resolved measurements enabled the generation of two-dimensional heat maps representing the distribution of refractive index variations across the tissue imprint. Heat maps provided a qualitative and spatial visualization of tissue heterogeneity and differences between tumor and peritumoral samples (**Figure S3C**).

- 1. **Refractive index distribution and summary metrics**

Beyond spatial visualization, RI values obtained from all measurement points within a given imprint were pooled for quantitative analysis. The distribution of RI values was represented using histograms, allowing comparison between tumor and peritumoral tissue samples (**Figure S3D**).

For statistical analysis and paired comparisons, a single representative value was derived for each sample. This value corresponded to the mean RI calculated from all measurement points within the active sensing area. The mean RI was used as the primary quantitative parameter for subsequent statistical analysis, as it provides a robust and practical summary of the optical response of each tissue sample.


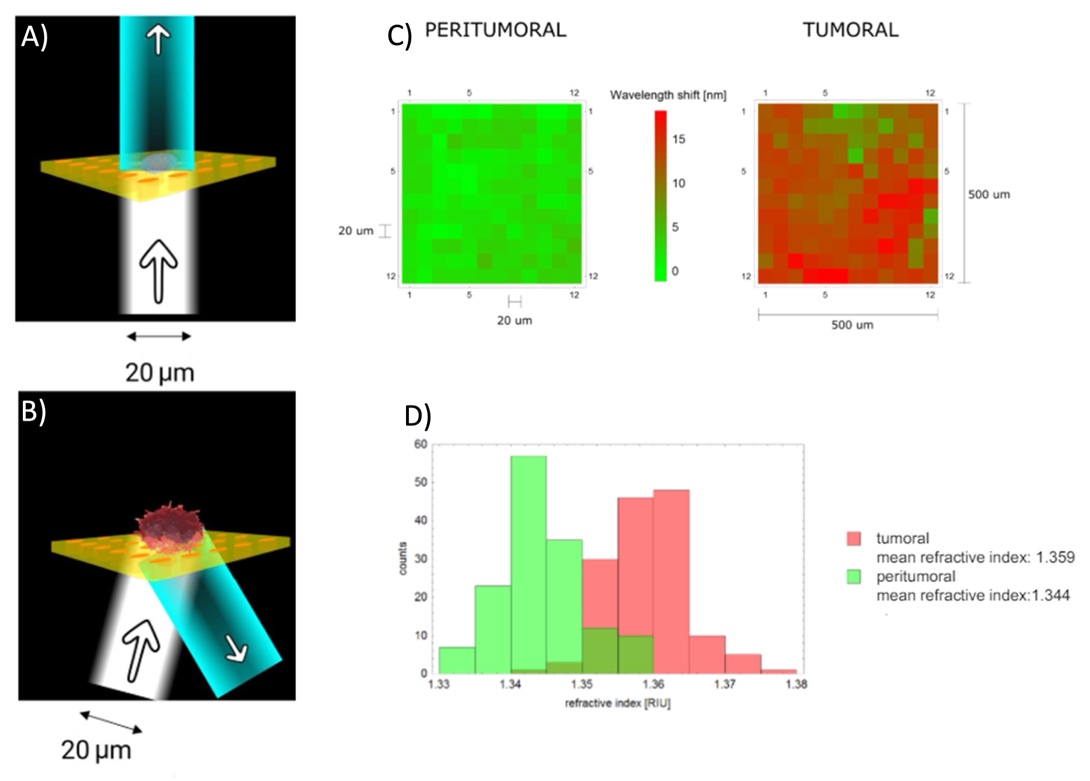


**Figure S3**. **Optical analysis modalities and data extraction from nanoplasmonic biosensor measurements.** **(A)** Schematic representation of transmission and **(B)** reflection measurement configurations for EOT-based sensing. **(C)** Spatial scanning of the nanohole array enables generation of refractive index heat maps across the active sensing area for peritumoral and tumor tissue imprints. **(D)** Aggregation of spatially resolved measurements allows construction of refractive index distributions and extraction of a mean refractive index value for each sample, which is used for quantitative comparison and statistical analysis.
